# Work–Family Behavioral Role Conflict and Turnover Intention Among Clinical Nurses: Multiple Mediating Effects of Role Overload, Presenteeism, and Psychological Capital

**DOI:** 10.1155/jonm/8648929

**Published:** 2026-08-13

**Authors:** Xiao Yue, Wanling Li, Quan Yuan, Chenxi Zhou, Rong Zhou, Mei Wang, Yu Liu

**Affiliations:** ^1^ Nursing Department, Tongji Hospital, Tongji Medical College, Huazhong University of Science and Technology, WuHan, China, hust.edu.cn; ^2^ Nursing Department, Affiliated Cancer Hospital of Zhengzhou University / Henan Cancer Hospital, Zhengzhou, China

**Keywords:** Conservation of Resources theory, nurses, presenteeism, psychological capital, role overload, turnover intention, work–family behavioral role conflict

## Abstract

**Background:**

Work–family behavioral role conflict (WFBRC) is an important stressor in the nursing work environment and may be associated with turnover intention. However, the roles of role overload, presenteeism, and (PsyCap) in this relationship have rarely been examined simultaneously among Chinese clinical nurses.

**Aims:**

Guided by the Conservation of Resources (COR) theory, this study aimed to examine the relationship between WFBRC and turnover intention among Chinese clinical nurses, as well as the mediating and moderating roles of role overload, presenteeism, and PsyCap.

**Methods:**

This was a multicenter cross‐sectional study. A total of 1225 registered nurses from 12 tertiary hospitals in China completed validated instruments assessing WFBRC, role overload, presenteeism, PsyCap, and turnover intention. Data were analyzed using descriptive statistics, Pearson correlation analysis, and bias‐corrected bootstrap analyses with 5000 resamples to test mediation and moderated mediation effects. Sensitivity analyses were conducted after controlling for demographic and occupational covariates.

**Results:**

WFBRC was positively associated with role overload, presenteeism, and turnover intention and negatively associated with PsyCap. Parallel mediation analyses showed that role overload, presenteeism, and PsyCap jointly mediated the association between WFBRC and turnover intention, with role overload showing the strongest indirect effect, followed by presenteeism, whereas PsyCap showed a smaller but significant indirect effect. A significant serial pathway through role overload and presenteeism was also identified. Moderated mediation analyses suggested that PsyCap may buffer the associations of role overload and presenteeism with turnover intention in separate second‐stage models; however, this pattern was not retained when both moderated pathways were entered simultaneously.

**Conclusion:**

WFBRC was significantly associated with nurses’ turnover intention through both direct and resource‐related pathways. Role overload and presenteeism were the primary mediators, whereas PsyCap showed a smaller indirect effect and limited buffering effects in selected models.

**Implications for Nursing Management:**

Organizations and nursing managers should address WFBRC proactively. Routine assessment of role overload and presenteeism can enable early detection of risk and timely intervention, while efforts to strengthen nurses’ PsyCap may further help break the resource‐loss process and support workforce retention.

## 1. Introduction

Globally, the nursing workforce continues to face unprecedented levels of occupational stress, workload pressure, and emotional exhaustion, contributing to a persistent shortage of nursing personnel [1, 2]. The International Council of Nurses recently reported that chronic understaffing, unsafe working conditions, and inadequate pay are major drivers of nurse burnout, attrition, and retention difficulties worldwide, with a current global shortage of 5.9 million nurses and an estimated need for 30 million additional nurses to meet future health care demands [3]. Nurse turnover imposes a substantial burden on healthcare systems by increasing recruitment costs, disrupting care continuity, and threatening patient safety [4–6]. Turnover intention, often regarded as the most immediate precursor of actual turnover behavior, may reflect nurses’ psychological withdrawal from their organizations and serves as an early indicator of potential workforce loss [4].

Among the psychosocial determinants of turnover intention, work–family behavioral role conflict (WFBRC), defined as the incompatibility between behavioral expectations from work and family domains, has emerged as a critical stressor that undermines nurses’ well‐being and increases psychological fatigue, presenteeism, and organizational retention [7–9]. Nurses frequently experience blurred boundaries between work and family responsibilities due to rotating shifts, long working hours, and emotional labor, which collectively amplify WFBRC [10–12]. In China, within a cultural context that emphasizes dedication, self‐sacrifice, collectivism, and strong family role obligations, nurses may be more likely to prioritize work demands over personal health and family needs [13, 14]. At the institutional level, nurse staffing shortages and concerns regarding professional recognition and effort–reward imbalance may further intensify work‐related strain [15]. In addition, clinical nurses often work in high‐intensity hospital settings characterized by heavy patient loads and sustained pressure related to care quality and patient safety, which gradually deplete their psychological resources and resilience [16, 17]. Under these circumstances, WFBRC may be particularly salient, underscoring the need to better understand its contribution to turnover intention in this population.

## 2. Background

Consistent with the Conservation of Resources (COR) theory, stress arises when individuals perceive a threat to their valued resources or fail to replenish them after loss [18, 19]. Within this theoretical framework, WFBRC can be understood as a resource threat and an ongoing source of resource loss, because nurses must continuously invest time, energy, and emotional resources to meet competing demands from both work and family roles [20, 21]. Prolonged exposure to WFBRC drains these personal reserves, leading to adverse outcomes such as role overload, reduced psychological capacity, increased turnover, and absence intentions [22, 23].

Role overload, characterized by an imbalance between work demands and the personal or organizational resources available to meet them, can be viewed as a proximal manifestation of resource depletion process [24]. An imbalance between excessive work demands and limited personal or organizational resources places nurses at a heightened risk of role overload, leading to chronic stress and reduced job satisfaction, both of which are strong predictors of turnover intention [25, 26]. Presenteeism represents another behavioral consequence of continued resource loss, referring to the tendency to remain at work despite illness or fatigue, often in an effort to protect professional identity or avoid guilt associated with absenteeism [27]. Presenteeism may initially appear to reflect dedication; it paradoxically exacerbates exhaustion, impairs performance, and accelerates resource depletion [28, 29].

In contrast, psychological capital (PsyCap) works as a protective and positive psychological resource comprising hope, self‐efficacy, resilience, and optimism [30]. Positive PsyCap reduces symptoms of anxiety among female nurses and helps them maintain motivation under pressure [31]. Accumulating evidence indicates that PsyCap enhances job satisfaction and reduces job burnout by restoring psychological resources eroded by occupational strain [31–33]. From a COR perspective, turnover intention may be understood as a downstream withdrawal response that emerges when resource loss persists and individuals perceive leaving the job as a way to protect their remaining resources.

Although previous studies have linked WFBRC to turnover intention, important gaps remain. Most existing research has examined these associations in a fragmented manner, focusing on isolated variables rather than a more integrated perspective on how WFBRC may be associated with turnover intention. In particular, the parallel and sequential roles of role overload, presenteeism, and PsyCap have rarely been examined together within a unified framework grounded in COR theory. These mechanisms also remain insufficiently explored among clinical nurses in the Chinese healthcare context, where work–family strain, workload pressure, and organizational constraints may make such processes especially salient. To address these gaps, the present study aimed to examine the associations between WFBRC and turnover intention among clinical nurses, as well as the potential mediating and moderating mechanisms involved, and to provide empirical evidence relevant to workforce sustainability in healthcare organizations. Accordingly, the following hypotheses were proposed: H1, WFBRC is positively associated with turnover intention among clinical nurses; H2, role overload, presenteeism, and PsyCap mediate the relationship between WFBRC and turnover intention; H3, WFBRC influences turnover intention through a serial mediating pathway involving role overload and presenteeism; and H4, PsyCap moderates the associations of role overload and presenteeism with turnover intention.

## 3. Methods

### 3.1. Setting and Sample

A multicenter cross‐sectional survey using convenience sampling was conducted between June and August 2025. Clinical nurses from 12 tertiary hospitals across six provinces and cities in China were invited to participate. The collected sample was distributed as follows: Wuhan, Hubei Province (*n* = 298, 23.88%); Taiyuan, Shanxi Province (*n* = 237, 18.99%); Zhengzhou, Henan Province (*n* = 201, 16.11%); Changsha, Hunan Province (*n* = 174, 13.94%); Jinan, Shandong Province (*n* = 160, 12.82%); and Hangzhou, Zhejiang Province (*n* = 178, 14.26%).

The following were the inclusion criteria for participants: (1) holding a valid nursing license and having at least 1 year of clinical work experience; (2) being able to independently complete the questionnaire; (3) currently engaged in direct patient care; and (4) voluntarily agreeing to participate and providing informed consent. The exclusion criteria were as follows: (1) student nurses, visiting nurses, or those in standardized training programs; (2) administrative nurses without direct patient care responsibilities; and (3) nurses who had been on extended leave, such as maternity, sick, or personal leave, for 3 months or longer.

The sample size was estimated utilizing the Monte Carlo Indirect Effect Power Analysis software [34]. A target power (1‐β) of 0.8 and a confidence interval (CI) of 95% (corresponding to *α* = 0.05) were set. A pilot study with a study population of 50 was conducted based on the above research methodology. Based on pilot study findings, the sample size calculation was derived from correlation values and standard deviations (SDs). Accounting for a dropout rate of 10%, the minimum required sample size for this study was determined to be 460 participants. Considering that structural equation modeling (SEM) generally requires a larger sample size, typically at least 10 times the number of measurement items [35], and that the present study included 75 items across all scales, the target sample size was set at ≥ 834 after accounting for a potential 10% missing rate.

### 3.2. Instrument

#### 3.2.1. Demographic and Work‐Related Characteristics

A brief self‐designed questionnaire was developed based on previous literature to collect participants’ demographic and occupational information [36–38], including department, gender, age, marital status, parental status, education level, professional title, job function, employment type, work stage, work experience, average weekly working hours, and average monthly night shifts. Professional title referred to the formal nursing rank within the Chinese professional title system (Junior nurse, Senior nurse, Nurse‐in‐charge, and Assistant chief senior nurse and above). Job function referred to the participant’s actual work role, including clinical nurse and clinical nurse with additional administrative, teaching, or research duties.

#### 3.2.2. Work–Family Behavioral Role Conflict Scale (WFBRC‐S)

WFBRC was measured using the WFBRC‐S [39]. The scale consists of two dimensions, work‐to‐family behavioral role conflict (Work‐to‐Family BRC) and family‐to‐work behavioral role conflict (Family‐to‐Work BRC), each containing 15 items. Participants rated the frequency of each behavior on a five‐point Likert scale ranging from 1 (never) to 5 (very frequently). The total WFBRC score was calculated as the sum of the two subdimensions, with higher scores indicating a greater frequency of behavioral role conflict between work and family. The Chinese version has been validated and shown good reliability and validity among Chinese clinical nurses (Cronbach’s *α* = 0.958) [40].

#### 3.2.3. Role Overload Scale

Role overload was assessed using the Role Overload Scale developed by Peterson, Smith, and Akande [41] and revised by Li et al. (Cronbach’s *α* = 0.900) [42]. The scale consists of five items with a single‐dimensional structure. Each item is rated on a five‐point Likert scale ranging from 1 (strongly disagree) to 5 (strongly agree), with no reverse scoring. Higher scores indicate a greater perceived role overload.

#### 3.2.4. Stanford Presenteeism Scale‐6 (SPS‐6)

Presenteeism due to health‐related issues was assessed using the Chinese version of SPS‐6 [43], translated and revised by Zhao et al. (Cronbach’s *α* = 0.760) [44]. The SPS‐6 consists of six items across two dimensions: work limitation (items 1–4) and work energy (items 5–6). Each item is rated on a five‐point Likert scale ranging from 1 (strongly disagree) to 5 (strongly agree). Items 5 and 6 are reverse scored, while the remaining items are positively scored. Total scores range from 6 to 30, with higher scores indicating greater productivity loss and more severe presenteeism.

#### 3.2.5. PsyCap Scale for Nurses

PsyCap was measured using the Psychological Capital Scale for Nurses (Cronbach’s *α* = 0.981) [45], which consists of six dimensions: hope, collaboration and communication, emotional intelligence, sense of responsibility, resilience, and self‐confidence, with a total of 30 items. Each item was rated on a six‐point Likert scale ranging from 1 (strongly disagree) to 6 (strongly agree). The total score was calculated as the sum of the six dimension scores and ranged from 30 to 180, with higher scores indicating a higher overall level of PsyCap among nurses. The overall Cronbach’s *α* of the scale was 0.981. In addition, confirmatory factor analysis showed that the overall measurement model was acceptable (*x* ^2^/df = 3.559, GFI = 0.724, CFI = 0.941, TLI = 0.933, NFI = 0.919, RMSEA = 0.087, RMR = 0.013), supporting the scale’s overall reliability and validity.

#### 3.2.6. Turnover Intention Scale

Turnover intention was measured using the Turnover Intention Scale developed by Wayne, Shore, and Liden [46]. The scale consists of four items; each item was rated on a five‐point Likert scale ranging from 1 (strongly disagree) to 5 (strongly agree). Higher scores indicate stronger turnover intention among employees. The Chinese version, validated by Zhang et al. [47], has demonstrated good reliability and validity. In the present study, the Cronbach’s *α* for this scale was 0.917.

### 3.3. Data Collection and Quality Control

The study used the Wenjuanxing software (https://www.wjx.cn/) for data collection. After obtaining approval from the nursing administrators of each participating hospital, two designated liaisons in each hospital invited clinical nurses to complete the online questionnaire privately during their nonworking hours by scanning a (Quick Response) QR code. To ensure consistency in data collection, all research assistants received standardized training before the survey. The questionnaire instructions included the study purpose, significance, and completion guidelines. All participants were required to read and confirm a unified informed consent statement prior to participation. The survey was completely anonymous, and confidentiality was strictly maintained. Each device or account was permitted to submit only one response, and only questionnaires with all items completed could be submitted. After data collection, two researchers independently reviewed the responses. Questionnaires were excluded if they met any of the following criteria: completion time shorter than 4 minutes (the shortest time observed during the pilot test), identical or patterned responses across all items, or logically inconsistent answers. This study was conducted and reported in accordance with the Strengthening the Reporting of Observational Studies in Epidemiology (STROBE) guidelines [48].

### 3.4. Statistical Analysis

Data were analyzed using Mplus version 8.3 and SPSS version 27.0. The normality of continuous variables was assessed through skewness and kurtosis coefficients [49, 50]. Variables with an approximately normal distribution were described as mean and SD, while categorical variables were presented as frequencies and percentages. Pearson’s correlation analysis was conducted to examine the relationships among major variables. The hypothesized sequential associations among WFBRC, role overload, presenteeism, PsyCap, and turnover intention were specified on the basis of COR theory and prior empirical literature. A parallel multiple mediation model was tested using SEM with maximum likelihood estimation in Mplus 8.3. In the model, WFBRC was specified as the independent variable; turnover intention as the dependent variable; and role overload, PsyCap, and presenteeism as latent mediating variables. The mediation and moderating effects were tested using a bias‐corrected bootstrap method with 5000 resamples to generate 95% CI for specific and total indirect effects. Effects were considered significant if the 95% CI did not include zero [51]. To ensure the robustness of the findings, sensitivity analyses were performed by controlling for demographic and occupational covariates, including age, gender, years of work experience, and department type. A two‐tailed *p* value of < 0.05 was considered statistically significant.

### 3.5. Ethical Considerations

This study was conducted in accordance with the ethical principles outlined in the Declaration of Helsinki. Ethical approval was obtained from the Institutional Review Board of the participating institution (Approval No. TJ‐IRB20230385) before data collection. Participation in the study was entirely voluntary. All participants were fully informed about the purpose, procedures, and their rights as research subjects. Informed consent was obtained electronically before participants accessed the questionnaire. The information provided clearly stated that participants could withdraw from the study at any time without any consequences. Anonymity and confidentiality of all responses were strictly maintained throughout the research process.

## 4. Results

### 4.1. Demographic and Professional Characteristics of Participants

A total of 1248 questionnaires were collected, and 1225 valid responses were retained after excluding those that did not meet the inclusion criteria, yielding an effective response rate of 98.16%. Table 1 presents the demographic and professional characteristics of the participants. Most participants were female (93.3%), aged 26–45 years (82.3%), and married (69.2%). The majority were contract employees and worked in clinical positions, and more than half reported 5–8 night shifts per month.

**TABLE 1:** Demographic and professional characteristics of participants (*N* = 1225).

Variables	*N* (%)
*Department*	
Internal medicine	235 (19.2)
Surgery	230 (18.8)
Gynecology and obstetrics	99 (8.1)
Pediatrics	85 (6.9)
Geriatrics	87 (7.1)
Oncology	67 (5.5)
Comprehensive department	95 (7.8)
Outpatient clinic	50 (4.1)
Emergency department	220 (18.0)
Other	57 (4.7)

*Gender*	
Male	82 (6.7)
Female	1143 (93.3)

*Age*	
≤ 25 years	135 (11.0)
26–35 years	625 (51.0)
36–45 years	383 (31.3)
≥ 45 years	82 (6.7)

*Marital status*	
Unmarried, divorced, or widowed	377 (30.8)
Married	848 (69.2)

*Parental status*	
No children	442 (36.1)
With children	783 (63.9)

*Education level*	
Bachelor’s degree	1159 (94.6)
Master’s degree or above	66 (5.4)

*Professional title*	
Junior nurse	131 (10.7)
Senior nurse	441 (36.0)
Nurse‐in‐charge	606 (49.5)
Assistant chief senior nurse and above	47 (3.8)

*Job function*	
Clinical nurse	884 (72.2)
Clinical nurse with additional administrative/teaching/research duties	341 (27.8)

*Employment type*	
Permanent staff	214 (17.5)
Contract staff	1011 (82.5)

*Work stage*	
Fixed in a specific department	1101 (89.9)
Rotation	124 (10.1)

*Work experience (years)*	
≤ 5	358 (29.2)
6–10	300 (24.5)
11–15	401 (32.7)
> 15	166 (13.6)

*Average weekly working hours (hours)*	
≤ 40	744 (60.7)
> 40	481 (39.3)

*Average monthly night shifts*	
0	177 (14.4)
1–4	221 (18.0)
5–8	665 (54.3)
≥ 8	162 (13.2)

### 4.2. Descriptive and Correlation Analysis

Supporting Information Table S1 presents the descriptive statistics and normality tests of the main study variables. The skewness (−0.53–0.28) and kurtosis (−0.61–1.53) values of all variables were within the acceptable range of ±1 and ± 2, indicating approximate normal distributions. The mean (SD) of WFBRC, role overload, PsyCap, presenteeism, and turnover intention were 79.57 ± 21.51, 11.22 ± 4.04, 138.78 ± 24.74, 15.99 ± 3.89, and 9.56 ± 4.05, respectively.

Table 2 shows the Pearson’s correlation coefficients among the major variables. WFBRC was positively correlated with role overload (*r* = 0.584, *p* < 0.001), presenteeism (*r* = 0.504, *p* < 0.001), and turnover intention (*r* = 0.612, *p* < 0.001) and negatively correlated with PsyCap (*r* = −0.427, *p* < 0.001). Both work‐to‐family BRC (*r* = 0.510, *p* < 0.001) and family‐to‐work BRC (*r* = 0.573, *p* < 0.001) were significantly associated with turnover intention. Both role overload (*r* = 0.532, *p* < 0.001) and presenteeism (*r* = 0.429, *p* < 0.001) were positively associated with turnover intention, whereas PsyCap showed a significant negative correlation (*r* = −0.286, *p* < 0.001). The correlation coefficients ranged from low to moderate, indicating no severe multicollinearity and providing a sound basis for subsequent mediation analysis.

**TABLE 2:** Correlations among work–family behavioral role conflict, role overload, PsyCap, presenteeism, and turnover intention.

Variable	WFBRC	Role overload	Psychological capital	Presenteeism	Turnover intention
1. WFBRC	1	0.584^∗∗∗^	−0.427^∗∗∗^	0.504^∗∗∗^	0.612^∗∗∗^
1‐1 Work‐to‐family BRC	0.890^∗∗∗^	0.543^∗∗∗^	−0.286^∗∗∗^	0.375^∗∗∗^	0.510^∗∗∗^
1‐2 Family‐to‐work BRC	0.876^∗∗∗^	0.487^∗∗∗^	−0.473^∗∗∗^	0.520^∗∗∗^	0.573^∗∗∗^
2. Role overload		1	−0.325^∗∗∗^	0.492^∗∗∗^	0.638^∗∗∗^
3. Psychological capital			1	−0.395^∗∗∗^	−0.405^∗∗∗^
4. Presenteeism				1	0.551^∗∗∗^
5. Turnover intention					1

^∗∗∗^ *p* < 0.001.

Supporting Table S2 further presents the univariate analysis of turnover intention across demographic and professional characteristics. Significant group differences in turnover intention were observed across department, age, marital status, parental status, professional title, job function, work experience, average weekly working hours, and average monthly night shifts (all *p* < 0.05).

### 4.3. Mediation and Moderating Effect Analysis

#### 4.3.1. Parallel Multiple Mediation Model

**FIGURE 1:**
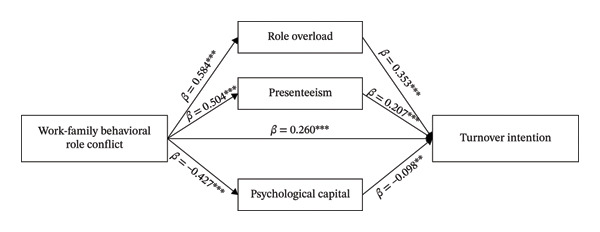
Parallel multiple mediation model of the relationship between work–family behavioral role conflict and turnover intention (Model 1).

Model 1 tested a parallel multiple mediation structure with role overload, PsyCap, and presenteeism specified as latent mediators between WFBRC and turnover intention, with standardized path coefficients presented in Figure 1 and Table 3 (model fit indices: *x* ^2^/df = 4.676, CFI = 0.967, TLI = 0.915, RMSEA = 0.068, and SRMR = 0.035). All paths reached statistical significance (*p* < 0.001). The total effect of WFBRC on turnover intention was 0.612, with a significant direct effect (*β = *0.260, *p* < 0.001). The total indirect effect was 0.352, accounting for 57.5% of the total effect. Among the three mediating paths, the indirect effect through role overload (Ind1) was the strongest (*β = *0.206, 95% CI [0.176, 0.241], *p* < 0.001), explaining 33.7% of the total effect. The mediating effect through presenteeism (Ind2) was also significant (*β = *0.104, 95% CI [0.080, 0.131], *p* < 0.001]), accounting for 17.0% of the total effect. The indirect effect through PsyCap (Ind3) was relatively weaker but significant (*β = *0.042, 95% CI [0.021, 0.065], *p* < 0.001), contributing 6.8% of the total effect. The model explained 53.8% of the variance in turnover intention (*R* ^2^ = 0.538).

**TABLE 3:** Parameter estimates of the direct and indirect paths in the parallel multiple mediation model of the relationship between work–family behavioral role conflict and turnover intention (Model 1).

Structural paths	*β*	95% CI
Total effect	0.612^∗∗∗^	(0.569, 0.661)
Direct effect (WFBRC ⟶ turnover intention)	0.260^∗∗∗^	(0.207, 0.315)
Total indirect effect	0.352^∗∗∗^	(0.311, 0.399)

*Direct paths*		
WFBRC ⟶ role overload	0.584^∗∗∗^	(0.538, 0.623)
WFBRC ⟶ psychological capital	−0.427^∗∗∗^	(−0.498, −0.350)
WFBRC ⟶ presenteeism	0.504^∗∗∗^	(0.464, 0.542)
Role overload ⟶ turnover intention	0.353^∗∗∗^	(0.297, 0.406)
Psychological capital ⟶ turnover intention	−0.098^∗∗∗^	(−0.150, −0.049)
Presenteeism ⟶ turnover intention	0.207^∗∗∗^	(0.151, 0.262)

*Indirect effects (mediation paths)*		
Ind1: WFBRC ⟶ role overload ⟶ turnover intention	0.206^∗∗∗^	(0.176, 0.241)
Ind2: WFBRC ⟶ presenteeism ⟶ turnover intention	0.104^∗∗∗^	(0.080, 0.131)
Ind3: WFBRC ⟶ psychological capital ⟶ turnover intention	0.042^∗∗∗^	(0.021, 0.065)

*Note: β* = standardized regression coefficient; CI = bias‐corrected bootstrap confidence interval. Effects were considered significant when the 95% CI did not include zero.

^∗∗∗^ *p* < 0.001.

#### 4.3.2. Serial Mediation Model

**FIGURE 2:**
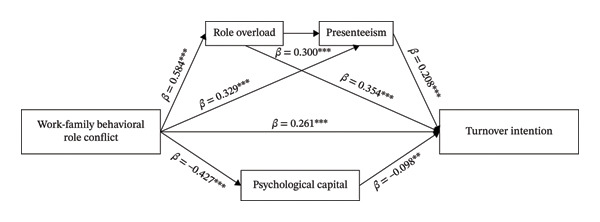
Serial mediation model of the relationship between work–family behavioral role conflict and turnover intention (Model 2).

Model 2 tested a serial mediation model linking WFBRC and turnover intention (model fit indices: *x* ^2^/df = 4.935, CFI = 0.975, TLI = 0.901, RMSEA = 0.071, SRMR = 0.038). A significant serial indirect pathway was identified, whereby WFBRC was associated with turnover intention through role overload and presenteeism in sequence (*β* = 0.036, 95% CI [0.026, 0.049], *p* < 0.001). This pathway accounted for 5.9% of the total effect. Detailed results are presented in Figure 2 and Supporting Table S3.

#### 4.3.3. Moderated Mediation Analysis

**FIGURE 3:**
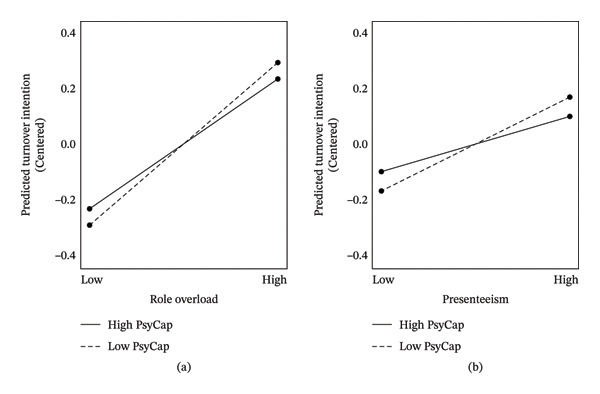
Simple slope plots of psychological capital’s moderating effects.

A series of moderated mediation models (Models 3–7) were conducted to examine whether PsyCap altered the indirect pathways from WFBRC to turnover intention via role overload and presenteeism. Supporting Information Figure S1 presents the first‐stage moderation models; PsyCap did not significantly moderate the association between WFBRC and role overload (*p* = 0.529, Model 3) or between WFBRC and presenteeism (*p* = 0.921, Model 4). Supporting Information Figure S2 presents second‐stage moderation models (Models 5–7), Supporting Information Table S4 presents the standardized structural path coefficients for Model 5, which tested the moderating effect of PsyCap on the pathway from role overload to turnover intention, and Supporting Information Table S5 summarizes the standardized structural path coefficients for Model 6, which examined the moderating effect of PsyCap on the pathway from presenteeism to turnover intention. Significant interaction effects were observed. PsyCap moderated the relationship between role overload and turnover intention (*β* = −0.058, *p* = 0.020; Model 5), as well as the association between presenteeism and turnover intention (*β* = −0.069, *p* = 0.004; Model 6). Conditional indirect effect analyses further revealed that, under low levels of PsyCap, the indirect effects of WFBRC on turnover intention through role overload and presenteeism were 0.192 and 0.119, respectively, whereas under high PsyCap, these effects decreased to 0.148 and 0.068, representing relative reductions of 23% and 43% (Figure 3). Supporting Information Table S6 presents the standardized structural path coefficients for Model 7, which examined the simultaneous moderating effects of PsyCap on both role overload and presenteeism. When both second‐stage moderation paths simultaneously entered into Model 7, neither interaction remained statistically significant.

#### 4.3.4. Sensitivity Analysis

To examine the robustness of the findings, a sensitivity analysis was performed by re‐estimating Model 1 while controlling for demographic and work‐related covariates, including age, gender, years of experience, and department type. Supporting Information Table S7 reports the standardized structural path coefficients for the parallel multiple mediation model with demographic and work‐related covariates controlled. The results showed that the direct and indirect effects remained significant and consistent in direction and magnitude, indicating the robustness of the mediation model.

In addition, supplementary multiple linear regression analyses were conducted to further examine whether the association between WFBRC and turnover intention differed across the two subdimensions of WFBRC (Supporting Information Table S8). In Model a, the total WFBRC score was positively associated with turnover intention (*β* = 0.600, *p* < 0.001). In Model b, when the two subdimensions entered simultaneously, both family‐to‐work BRC (*β* = 0.400, *p* < 0.001) and work‐to‐family BRC (*β* = 0.279, *p* < 0.001) were positively associated with turnover intention.

## 5. Discussion

The primary aim of this study was to examine whether WFBRC was associated with turnover intention among clinical nurses through multiple resource‐related factors, guided by COR theory. The findings extend current knowledge by showing that WFBRC was associated with turnover intention both directly and indirectly through parallel mediating pathways involving role overload, presenteeism, and PsyCap. In addition, a significant serial pathway linked WFBRC with turnover intention through role overload and presenteeism in sequence. The moderated mediation analyses further suggested that PsyCap may have a limited buffering role in the associations of role overload and presenteeism with turnover intention in separate second‐stage models, although this pattern was not retained when both second‐stage moderated pathways were tested simultaneously. Overall, these findings suggest that COR theory provides a useful interpretive framework and that role overload, presenteeism, and PsyCap may help explain the association between WFBRC and turnover intention among clinical nurses.

Our study further confirmed a significant positive direct effect of WFBRC on turnover intention. This result aligns with earlier studies showing that WFBRC is linked to negative professional retention outcomes, such as reduced work engagement, increased presenteeism, and stronger turnover intention [9, 52, 53]. China’s unique sociocultural expectations of caregiving responsibilities further intensify the conflict, particularly for married and child‐rearing female nurses, who represent a large proportion of the workforce [38]. Moreover, rotating shifts, night duties, and work demands extending into nonworking hours disrupt normal family routines and intensify the guilt and stress tied to family responsibilities that nurses are unable to fulfill [28]. Over time, these challenges reshape nurses’ career evaluations, shifting their priority from professional dedication to personal well‐being, leading them choose to leave rather than continue sacrificing family functioning [54]. Taken together, our findings underscore that WFBRC is not merely a work–life balance issue but a critical retention challenge rooted in structural and cultural dynamics.

Role overload is confirmed as the strongest mediator linking WFBRC to turnover intention. From a COR perspective, this pattern is consistent with the theoretical proposition that strain in one domain may be accompanied by broader resource‐related difficulties across domains [18]. WFBRC heightens perceived workload burden as strain accumulates and personal resources become insufficient to meet escalating job expectations [55, 56]. Nurses who repeatedly invest emotional and time resources to meet competing role expectations may feel increasingly unable to cope with escalating clinical responsibilities, thereby heightening role overload [57]. This pattern is consistent with previous research identifying excessive workload and inadequate staffing as major predictors of emotional exhaustion, declining job satisfaction, and turnover intention [26]. In China, nurses frequently take on multiple responsibilities, and policy changes in response to increasing healthcare demands means that nurses are under pressure to change their roles to be ever more effective [58, 59]. Importantly, role overload may reflect not only workload volume but also limited autonomy and insufficient organizational support [60].

Presenteeism also served as a significant mediator between WFBRC and turnover intention. When nurses continue working despite fatigue, illness, or psychological strain, they may place additional strain on already limited resources, which is consistent with a COR‐based interpretation [18]. This aligns with evidence that presenteeism often arises when work and nonwork demands conflict, reflecting pressure to maintain performance despite reduced functioning [61]. In high‐intensity clinical environments, nurses may feel compelled to remain present to avoid burdening colleagues, compromising patient safety, or receiving negative evaluations [62]. However, this seemingly adaptive behavior contributes to diminished productivity, emotional disengagement, and eventually heightened turnover intention [52, 63]. Further, an SEM study in the United States demonstrated that poor work environments, perceived stress, and work–life imbalance significantly increased presenteeism, which in turn led to missed care and burnout [64]. These findings converge to suggest that presenteeism in nursing may reflect not only individual coping struggles but also broader organizational system failures. Together, our study reinforces that reducing presenteeism is an essential workforce retention.

PsyCap demonstrated a relatively weaker but significant mediating effect in the link between WFBRC and turnover intention. This is consistent with prior evidence showing that PsyCap mediates the relationship between work–family conflict and turnover intention among Chinese nurses [65] and is associated with lower burnout, reduced anxiety, and better work‐related outcomes in nursing populations [66]. However, the moderating analyses suggested that PsyCap had a limited buffering role, and this pattern should be interpreted cautiously because it was not retained when both moderated pathways were considered simultaneously. Although previous research suggests that PsyCap may help delay withdrawal intention, it may be less able to offset multiple concurrent stress‐related pathways [67]. Recent evidence further suggests that nurses’ psychological resources are closely shaped by organizational conditions such as leadership, decent work perception, and workplace support [65, 68]. This may be especially true in Chinese hospitals, where heavy workloads, employment insecurity, and effort–reward imbalance may coexist, limiting the capacity of individual psychological resources to counterbalance structural and systemic stressors that may accumulate over time and ultimately lead nurses to leave the profession [69]. Therefore, strengthening PsyCap remains valuable, but its protective effect is likely to be maximized only when combined with organizational reforms aimed at reducing excessive demands, improving support, and creating a more sustainable practice environment.

Our study further identified a significant serial mediation pathway linking WFBRC to turnover intention through role overload and presenteeism, providing empirical support for the resource loss spiral proposed by the COR theory [18]. This finding extends the nursing literature by integrating several relationships that have often been examined separately. Previous studies have shown that work stressors are positively associated with presenteeism, whereas work resources are negatively associated with it, and that presenteeism may accelerate exhaustion, impair performance, and increase turnover intention [63]. Consistent with prior studies showing that workload strain often precedes chronic fatigue, productivity loss, and turnover among clinical nurses [70, 71], our findings suggest that WFBRC may intensify role overload, which in turn increases the likelihood of presenteeism and ultimately turnover intention. This pathway may be particularly salient in nursing, especially in the Chinese clinical context, where staffing shortages, sustained patient‐care demands, expanding nonclinical responsibilities, and strong professional responsibility, together with cultural expectations of dedication and endurance, may make nurses more likely to continue working despite strain [72, 73]. If organizations fail to provide timely support, nurses may view leaving the job as the only viable strategy to halt ongoing resource exhaustion [72]. Therefore, interventions should aim to interrupt this loss process early by reducing overload and addressing the conditions that normalize presenteeism.

## 6. Implications for Nurse Managers

These findings highlight several actionable directions for healthcare organizations. First, managing workload should be prioritized through staffing optimization, task redistribution, and scheduling reforms to reduce role overload and WFBRC. Second, organizations must actively prevent presenteeism by fostering a supportive attendance culture, enhancing access to occupational health resources, and ensuring adequate backup staffing. Third, interventions aimed at strengthening nurses’ PsyCap, such as resilience training, positive coaching, and peer support programs, can support recovery of internal resources and reduce turnover tendencies. Leadership involvement is essential to create a resource‐enriching environment where nurses feel valued and supported. By addressing both structural and psychological risk mechanisms, hospitals can reduce turnover intention, improve nurse well‐being, and ultimately enhance care quality and workforce sustainability.

## 7. Limitations and Future Directions

Several limitations should be acknowledged. First, the cross‐sectional design prevents conclusions about causal relationships and does not allow the temporal ordering implied in the mediation and moderated mediation models to be empirically verified, indicating the need for longitudinal and intervention studies to verify temporal mechanisms. Second, because all variables were measured using self‐reported questionnaires, reporting bias and common method bias could not be completely excluded, although procedural and statistical remedies were used to reduce their potential influence. Third, although the sample was large and multicentered, all participants were recruited from tertiary hospitals in China, which may limit generalizability to other healthcare settings or cultural contexts. Data were collected between June and August 2025, and potential seasonal variation in nurses’ workload could not be fully ruled out, although work‐related factors such as department, weekly working hours, and night shifts were considered in the analyses. Additionally, other relevant COR variables such as organizational justice, leadership style, and coping strategies were not included but may further shape the observed relationships. Future research should incorporate multisource data, examine contextual moderators, account for potential seasonal variation in workload, and develop resource‐enhancing interventions targeting both individual PsyCap and organizational work design.

## 8. Conclusion

This multicenter cross‐sectional study showed that WFBRC was significantly associated with turnover intention among clinical nurses, both directly and indirectly through role overload, presenteeism, and PsyCap. Role overload and presenteeism emerged as the primary mediators, whereas PsyCap showed a smaller indirect effect and limited buffering effects in selected models. These findings underscore that simply improving individual resilience is insufficient unless organizational sources of conflict and workload are addressed. Effective workforce retention requires integrated strategies that reduce role overload, prevent presenteeism, and simultaneously strengthen nurses’ psychological resources. By enhancing resource protection and recovery, healthcare institutions may better sustain a stable and motivated nursing workforce.

## Funding

This work was supported by Natural Science Foundation of Hubei Province (grant number 2024AFB903); the 2024 Nursing Research Fund of Tongji Hospital, Tongji Medical College, and Huazhong University of Science and Technology (grant number 2024D07).

## Disclosure

Patient or Public Contribution: No patients, service users, caregivers, or members of the public were involved in the design, conduct, analysis, or reporting.

## Conflicts of Interest

The authors declare no conflicts of interest.

## Supporting Information

Additional supporting information can be found online in the Supporting Information section.

## Supporting information


**Supporting Information** Table S1: Descriptive statistics with normality test. Table S2: Univariate analysis of turnover intention scores across demographic and professional characteristics of participants (*N* = 1225). Table S3: Parameter estimates of the direct and indirect paths in the serial multiple mediation model of the relationship between work–family behavioral role conflict and turnover intention (Model 2). Figure S1: First‐stage moderation models. Figure S2: Second‐stage moderation models. Table S4: Standardized structural path coefficients with moderating effect of psychological capital on role overload (Model 5). Table S5: Standardized structural path coefficients with moderating effect of psychological capital on presenteeism (Model 6). Table S6: Standardized structural path coefficients with moderating effect of psychological capital on both role overload and presenteeism (Model 7). Table S7: Standardized structural path coefficients of the parallel multiple mediation model with demographic and work‐related covariates controlled (Model 1 with covariates). Table S8: Multiple linear regression analyses of turnover intention using the total WFBRC score and its two subdimensions (*N* = 1225).

## Data Availability

The data that support the findings of this study are available from the corresponding author upon reasonable request.

## References

[1] Bahlman‐van Ooijen W. , Malfait S. , Huisman‐de Waal G. , and Hafsteinsdóttir T. B. , Nurses’ Motivations to Leave the Nursing Profession: A Qualitative Meta‐Aggregation, Journal of Advanced Nursing. (2023) 79, no. 12, 4455–4471, 10.1111/jan.15696. 37209086

[2] Petersen J. , Wendsche J. , and Melzer M. , Nurses’ Emotional Exhaustion: Prevalence, Psychosocial Risk Factors and Association to Sick Leave Depending on Care setting—A Quantitative Secondary Analysis, Journal of Advanced Nursing. (2023) 79, no. 1, 182–193, 10.1111/jan.15471. 36281066

[3] International Council of Nurses , International Nurses Day 2025: Caring for Nurses Strengthens Economies, 2025, International Council of Nurses, Geneva, Switzerland, https://www.icn.ch/sites/default/files/2025-04/ICN_IND2025_report_EN_A4_FINAL_0.pdf.

[4] Ali S. I. and Shaban M. , The Mediating Effect of Emotional Intelligence on the Association Between Gender Role Conflict and Turnover Intentions in Nursing Practice, Journal of Advanced Nursing. (2025) 82, no. 6, 6636–6647, 10.1111/jan.70305. 41117633

[5] Bae S. , Noneconomic and Economic Impacts of Nurse Turnover in Hospitals: A Systematic Review, International Nursing Review. (2022) 69, no. 3, 392–404, 10.1111/inr.12769. 35654041 PMC9545246

[6] Sasso L. , Bagnasco A. , Catania G. et al., Push and Pull Factors of Nurses’ Intention to Leave, Journal of Nursing Management. (2019) 27, no. 5, 946–954, 10.1111/jonm.12745. 30614593

[7] Figueiredo A. R. , Gaspar F. , Baixinho C. , and Lucas P. , How the Nursing Practice Environment Influences Retention and Turnover Intention: An Umbrella Review Protocol, Nursing Reports. (2024) 14, no. 4, 3233–3241, 10.3390/nursrep14040235. 39585126 PMC11587427

[8] Halter M. , Boiko O. , Pelone F. et al., The Determinants and Consequences of Adult Nursing Staff Turnover: A Systematic Review of Systematic Reviews, BMC Health Services Research. (2017) 17, no. 1, 10.1186/s12913-017-2707-0. PMC5732502 29246221

[9] Yildiz B. , Yildiz H. , and Ayaz Arda O. , Relationship Between work–family Conflict and Turnover Intention in Nurses: A Meta‐Analytic Review, Journal of Advanced Nursing. (2021) 77, no. 8, 3317–3330, 10.1111/jan.14846. 33855744

[10] Leineweber C. , Chungkham H. S. , Westerlund H. , Tishelman C. , and Lindqvist R. , Hospital Organizational Factors Influence work–family Conflict in Registered Nurses: Multilevel Modeling of a Nation-wide cross-sectional Survey in Sweden, International Journal of Nursing Studies. (2014) 51, no. 5, 744–751, 10.1016/j.ijnurstu.2013.09.010. 24144276

[11] Safieh S. , Shochat T. , and Srulovici E. , The Mediating Role of Sleep Quality in the Relationship Between Quick-Return Shift Work Schedules and Work–Family Conflict: A Cross-Sectional Study, Journal of Nursing Research. (2025) 33, no. 2, 10.1097/jnr.0000000000000663. 40063001

[12] Singh C. , Cross W. , Munro I. , and Jackson D. , Occupational Stress Facing Nurse academics—A Mixed‐Methods Systematic Review, Journal of Clinical Nursing. (2020) 29, no. 6, 720–735, 10.1111/jocn.15150. 31856356

[13] Zhang X. , Wei N. , Li M. et al., Sickness Presenteeism, Job Burnout, Social Support and health-related Productivity Loss Among Nurses in the Chinese Nurses’ Health Cohort Study (TARGET): A cross-sectional Survey, International Journal of Nursing Studies. (2025) 162, 10.1016/j.ijnurstu.2024.104962. 39615431

[14] Lui J. N. M. , Andres E. B. , and Johnston J. M. , How do Organizational Culture and Leadership Style Affect Nurse Presenteeism and Productivity?: A Cross Sectional Study of Hong Kong Acute Public Hospitals, International Journal of Nursing Studies. (2024) 152, 10.1016/j.ijnurstu.2023.104675. 38277926

[15] Liu J. , Zheng J. , Liu K. et al., Workplace Violence Against Nurses, Job Satisfaction, Burnout, and Patient Safety in Chinese Hospitals, Nursing Outlook. (2019) 67, no. 5, 558–566, 10.1016/j.outlook.2019.04.006. 31202444

[16] Yuan L. , Li Y. , Yan H. et al., Effects of work-family Conflict and Anxiety in the Relationship Between work-related Stress and Job Burnout in Chinese Female Nurses: A Chained Mediation Modeling Analysis, Journal of Affective Disorders. (2023) 324, 309–316, 10.1016/j.jad.2022.12.112. 36586602

[17] Chen X. , Li Q. , Xu F. , and Han B. , The Mediating Role of Resilience Between work-family Conflict and Career Development Among Chinese Nurses: A cross-sectional Study, Journal of Nursing Management. (2021) 29, no. 6, 1733–1741, 10.1111/jonm.13323. 33797812

[18] Hobfoll S. E. and Hou W. K. , Conservation of Resources Theory and Traumatic Stress Placed in the Context of Meaning-Making, The Routledge International Handbook of Human Significance and Mattering, 2025, Routledge, New York, 289–301, 10.4324/9781003424437-27.

[19] Prapanjaroensin A. , Patrician P. A. , and Vance D. E. , Conservation of Resources Theory in Nurse Burnout and Patient Safety, Journal of Advanced Nursing. (2017) 73, no. 11, 2558–2565, 10.1111/jan.13348. 28543427

[20] Grandey A. A. and Cropanzano R. , The Conservation of Resources Model Applied to Work–Family Conflict and Strain, Journal of Vocational Behavior. (1999) 54, no. 2, 350–370, 10.1006/jvbe.1998.1666.

[21] Liao E. Y. , Lau V. P. , Hui R. T. , and Kong K. H. , A Resource-based Perspective on work–family Conflict: Meta-Analytical Findings, Career Development International. (2019) 24, no. 1, 37–73, 10.1108/CDI-12-2017-0236.

[22] Karatepe O. M. and Karadas G. , The Effect of Psychological Capital on Conflicts in the work–family Interface, Turnover and Absence Intentions, International Journal of Hospitality Management. (2014) 43, 132–143, 10.1016/j.ijhm.2014.09.005.

[23] Kim L. , Maijan P. , and Yeo S. F. , Spillover Effects of work–family Conflict on Job Consequences Influencing Work Attitudes, Scientific Reports. (2025) 15, no. 1, 10.1038/s41598-025-93940-3. PMC11914499 40097660

[24] Brown S. P. , Jones E. , and Leigh T. W. , The Attenuating Effect of Role Overload on Relationships Linking Self-Efficacy and Goal Level to Work Performance, Journal of Applied Psychology. (2005) 90, no. 5, 972–979, 10.1037/0021-9010.90.5.972. 16162069

[25] Jourdain G. and Chênevert D. , Job demands–resources, Burnout and Intention to Leave the Nursing Profession: A Questionnaire Survey, International Journal of Nursing Studies. (2010) 47, no. 6, 709–722, 10.1016/j.ijnurstu.2009.11.007. 20138278

[26] Wong K. P. , Zhang B. , Xie Y. J. et al., Impacts of Job Demands on Turnover Intention Among Registered Nurses in Hong Kong Public Hospitals: Exploring the Mediating Role of Burnout and Moderating Effect of Pay Level Satisfaction, Journal of Nursing Management. (2024) 2024, no. 1, 10.1155/2024/3534750. PMC11918518 40224872

[27] Miraglia M. and Johns G. , Going to Work Ill: A meta-analysis of the Correlates of Presenteeism and a dual-path Model, Journal of Occupational Health Psychology. (2016) 21, no. 3, 261–283, 10.1037/ocp0000015. 26550958

[28] Choi W.-S. , Kang S.-W. , and Choi S. B. , The Dark Side of Mobile Work During Non-work Hours: Moderated Mediation Model of Presenteeism Through Conservation of Resources Lens, Frontiers in Public Health. (2024) 12, no. 2024, 10.3389/fpubh.2024.1186327. PMC10909990 38439760

[29] Fan S. , Zhou S. , Ma J. , An W. , Wang H. , and Xiao T. , The Role of the Nursing Work Environment, Head Nurse Leadership and Presenteeism in Job Embeddedness Among New Nurses: A cross-sectional Multicentre Study, BMC Nursing. (2024) 23, no. 1, 10.1186/s12912-024-01823-1. PMC10913553 38443951

[30] Xue B. , Wang S. , Chen D. , Hu Z. , Feng Y. , and Luo H. , Moral Distress, Psychological Capital, and Burnout in Registered Nurses, Nursing Ethics. (2024) 31, no. 3, 388–400, 10.1177/09697330231202233. 37737144

[31] Zhou J. , Yang Y. , Qiu X. et al., Serial Multiple Mediation of Organizational Commitment and Job Burnout in the Relationship Between Psychological Capital and Anxiety in Chinese Female Nurses: A cross-sectional Questionnaire Survey, International Journal of Nursing Studies. (2018) 83, 75–82, 10.1016/j.ijnurstu.2018.03.016. 29702357

[32] Kuhlmann R. and Süß S. , The Dynamic Interplay of Job Characteristics and Psychological Capital with Employee Health: A Longitudinal Analysis of Reciprocal Effects, Journal of Occupational Health Psychology. (2024) 29, no. 1, 14–29, 10.1037/ocp0000368. 38059985

[33] Santos A. , Chambel M. J. , and Castanheira F. , Wellbeing Among Hospital Nurses: A cross-sectional Study of the Contributions of Relational Job Characteristics, International Journal of Nursing Studies. (2020) 105, 10.1016/j.ijnurstu.2019.103438. 32200098

[34] Schoemann A. M. , Boulton A. J. , and Short S. D. , Determining Power and Sample Size for Simple and Complex Mediation Models, Social Psychological and Personality Science. (2017) 8, no. 4, 379–386, 10.1177/1948550617715068.

[35] Wolf E. J. , Harrington K. M. , Clark S. L. , and Miller M. W. , Sample Size Requirements for Structural Equation Models: An Evaluation of Power, Bias, and Solution Propriety, Educational and Psychological Measurement. (2013) 73, no. 6, 913–934, 10.1177/0013164413495237. PMC4334479 25705052

[36] Bae S.-H. , Comprehensive Assessment of Factors Contributing to the Actual Turnover of Newly Licensed Registered Nurses Working in Acute Care Hospitals: A Systematic Review, BMC Nursing. (2023) 22, no. 1, 10.1186/s12912-023-01190-3. PMC9899133 36739408

[37] Fei Y. , Jiang N. , Zhao H. , Zhang F. , Fu W. , and Yin X. , How work-family Conflict Influences Emergency Department Nurses’ Turnover Intention: The Mediating Role of Positive and Negative Affect, International Emergency Nursing. (2023) 68, no. 2023, 10.1016/j.ienj.2023.101289. 37087968

[38] Yao X. , Wen S. , Song Z. , Wang J. , Shen Y. , and Huang X. , Work–Family Conflict Categories and Support Strategies for Married Female Nurses: A Latent Profile Analysis, Frontiers in Public Health. (2024) 12, 10.3389/fpubh.2024.1324147. PMC10958783 38525344

[39] Clark M. A. , Early R. J. , Baltes B. B. , and Krenn D. , Work-Family Behavioral Role Conflict: Scale Development and Validation, Journal of Business and Psychology. (2019) 34, no. 1, 39–53, 10.1007/s10869-017-9529-2.

[40] Sun W. , Wu Y. , and Gao J. , Reliability and Validity of Chinese Version of the Work-Family Behavioral Role Conflict Scale, Chinese Journal of Nursing. (2023) 58, no. 14, 1787–1793, 10.3761/j.issn.0254-1769.2023.14.017.

[41] Peterson M. F. , Smith P. B. , Akande A. et al., Role Conflict, Ambiguity, And Overload: A 21-Nation Study, Academy of Management Journal. (1995) 38, no. 2, 429–452, 10.2307/256687.

[42] Li C. and Zhang Y. , The Effects of Role Stressors on Physical Health and Mental Health Among Chinese Teachers, Psychological Development and Education. (2009) 25, no. 1, 114–119.

[43] Koopman C. , Pelletier K. R. , Murray J. F. et al., Stanford Presenteeism Scale: Health Status and Employee Productivity, Journal of Occupational and Environmental Medicine. (2002) 44, no. 1, 14–20, 10.1097/00043764-200201000-00004. 11802460

[44] Zhao F. , Dai J. , and Yan S. , Reliability and Validity of the Chinese Version of the Stanford Presenteeism Scale (SPS-6), Chinese Journal of Industrial Hygiene and Occupational Diseases. (2010) 28, no. 9, 679–682, 10.3760/cma.j.issn.1001-9391.2010.09.012. 21126483

[45] Yuan Z. , Wang J. , and Jin M. , Development and Reliability and Validity of a Psychological Capital Scale for Nurses, Chinese Journal of Nursing. (2023) 58, no. 1, 74–80, 10.3761/j.issn.0254-1769.2023.01.010.

[46] Wayne S. J. , Shore L. M. , and Liden R. C. , Perceived Organizational Support and Leader-Member Exchange: A Social Exchange Perspective, Academy of Management Journal. (1997) 40, no. 1, 82–111, 10.2307/257021.

[47] Zhang K. , Tang N. , and Yin K. , The Relationship Between Turnover Intention and Behavioral Performance: The Moderating Effects of self-efficacy and Proactive Personality, Journal of Management Science. (2018) 31, no. 6, 117–127, 10.3969/j.issn.1672-0334.2018.06.009.

[48] Benchimol E. I. and Langan S. M. , STROBE Reporting Guidelines for Health Equity, BMJ. (2025) 390, 10.1136/bmj.r1079. 40903036

[49] George D. and Mallery P. , IBM SPSS Statistics 26 Step by Step: A Simple Guide and Reference, 2019, Routledge, New York, 10.4324/9780429056765.

[50] Kline R. B. , Principles and Practice of Structural Equation Modeling, 2023, The Guilford Press, London, https://www.guilford.com/books/Principles-and-Practice-of-Structural-Equation-Modeling/Rex-Kline/9781462551910.

[51] Hayes A. F. , Introduction to Mediation, Moderation, and Conditional Process Analysis: A Regression-Based Approach, 2022, The Guilford Press, New York, https://www.guilford.com/books/Introduction-to-Mediation-Moderation-and-Conditional-Process-Analysis/Andrew-Hayes/9781462549030.

[52] Gillet N. , Austin S. , Fernet C. et al., Workaholism, Presenteeism, work-family Conflicts and Personal and Work Outcomes: Testing a Moderated Mediation Model, Journal of Clinical Nursing. (2021) 30, no. 20, 2842–2853, 10.1111/jocn.15791. 33870550

[53] Huaman N. , Morales-García W. C. , Castillo-Blanco R. et al., An Explanatory Model of Work-family Conflict and Resilience as Predictors of Job Satisfaction in Nurses: The Mediating Role of Work Engagement and Communication Skills, Journal of Primary Care & Community Health. (2023) 14, 10.1177/21501319231151380. PMC9893370 36718818

[54] Bian X. and Mohd Sukor M. S. , The Mediating Effect of work-life Balance on the Relationship Between work-family Conflict and Psychological well-being Among Chinese Working Women, Scientific Reports. (2024) 14, no. 1, 10.1038/s41598-024-79322-1. PMC11550461 39521919

[55] McNeill I. M. and Cullington E. , Workload, Work‐Life Conflict, and Stress Amongst Mental Health Professionals: The Moderating Role of Segmentation Preference, Stress and Health. (2025) 41, no. 4, 10.1002/smi.70095. PMC12309875 40737200

[56] Wang J. , Zhao S. , Tong X. , Wang M. , and Wang Y. , Work–Family Conflict Among Primary Health Workers During the COVID ‐19 Pandemic: Its Mediating Role in the Relationship Between Workload and Job Burnout, Journal of Clinical Nursing. (2024) 33, no. 10, 3933–3942, 10.1111/jocn.17035. 38284495

[57] Dall’Ora C. , Ball J. , Reinius M. , and Griffiths P. , Burnout in Nursing: A Theoretical Review, Human Resources for Health. (2020) 18, no. 1, 10.1186/s12960-020-00469-9. PMC7273381 32503559

[58] Pang X. , Pan J. , Chen Z. , Cao X. , Shi J. , and Mao L. , Development and Validation of a Backpropagation Neural Network Model for Predicting Nursing Unit Staffing Needs: A cross-sectional Study, International Journal of Nursing Studies. (2025) 172, 10.1016/j.ijnurstu.2025.105207. 40972497

[59] Yu J. , Song H. , Shi H. , and Wang K. , Association Between work–family Conflict and Overall Well‐Being Among Chinese Nurse Leaders, Journal of Nursing Management. (2020) 28, no. 7, 1498–1503, 10.1111/jonm.13084. 32629527

[60] Alnazly E. , Allari R. , Alshareef B. , and Abu Al-khair F. , Analyzing Role Overload, Mental Health, and Quality of Life Among Jordanian Female Healthcare Professionals: A Cross-Sectional Study, International Journal of Women’s Health. (2023) 15, 1917–1930, 10.2147/IJWH.S435857. PMC10705508 38077235

[61] Freeling M. , Rainbow J. G. , and Chamberlain D. , Painting a Picture of Nurse Presenteeism: A multi-country Integrative Review, International Journal of Nursing Studies. (2020) 109, 10.1016/j.ijnurstu.2020.103659. 32585449

[62] Rainbow J. G. , Dudding K. M. , and Bethel C. , A Qualitative Study Describing Nurses’ Experiences with Presenteeism, The Journal of Nursing Administration: The Journal of Nursing Administration. (2021) 51, no. 3, 135–140, 10.1097/NNA.0000000000000984. 33570370

[63] Lui J. N. M. , Andres E. B. , and Johnston J. M. , Presenteeism Exposures and Outcomes Amongst Hospital Doctors and Nurses: A Systematic Review, BMC Health Services Research. (2018) 18, no. 1, 10.1186/s12913-018-3789-z. PMC6299953 30567547

[64] Rainbow J. G. , Gilbreath B. , and Steege L. M. , Risky Business: A Mediated Model of Antecedents and Consequences of Presenteeism in Nursing, Nursing Research. (2021) 70, no. 2, 85–94, 10.1097/NNR.0000000000000484. 33630531

[65] Yang Z.-Y. , Du Y. , Plummer V. , Hu X. , Wang A.-N. , and Guo Y.-F. , Work-Family Conflict and Turnover Intention of Nurses: The Mediating Role of Psychological Capital and the Moderating Role of Transformational Leadership, Contemporary Nurse. (2025) 61, no. 6, 573–583, 10.1080/10376178.2025.2553572. 40932421

[66] Hu H. , Chang S. , Tian G. et al., The Impact of Perceived Stress on Job Satisfaction Among Nurse Managers: A Moderated Mediation Model of Job Burnout and Psychological Capital, BMC Nursing. (2025) 24, no. 1, 10.1186/s12912-025-03607-7. PMC12278657 40691576

[67] Pyhäjärvi D. and Söderberg C. B. , The Straw that Broke the Nurse’s back-Using Psychological Contract Breach to Understand Why Nurses Leave, Journal of Advanced Nursing. (2024) 80, no. 12, 4989–5002, 10.1111/jan.16143. 38444207

[68] Zheng N. , Zhang T. , He X.-F. , and Zhu X.-Q. , Decent Work Perception and Its Relationship with Work-Related Flow and Psychological Capital Among Nurses: A Cross-Sectional Study, Journal of Advanced Nursing. (2026) 82, no. 4, 2947–2958, 10.1111/jan.17100. 40538106

[69] Cui Q. , Jiang J. , Zhong Y. , Du R. , Zhang Y. , and Ge Y. , The Systemic Roots of Chinese Nurse Burnout: A mixed-methods Study Using Natural Language Processing of Social Media Data, International Journal of Nursing Studies. (2026) 175, 10.1016/j.ijnurstu.2025.105324. 41500186

[70] Galanis P. , Moisoglou I. , Katsiroumpa A. et al., Workload Increases Nurses’ Quiet Quitting, Turnover Intention, and Job Burnout: Evidence from Greece, AIMS Public Health. (2025) 12, no. 1, 44–55, 10.3934/publichealth.2025004. 40248406 PMC11999808

[71] Huang H. , Liu L. , Yang S. , Cui X. , Zhang J. , and Wu H. , Effects of Job Conditions, Occupational Stress, and Emotional Intelligence on Chronic Fatigue Among Chinese Nurses: A cross-sectional Study, Psychology Research and Behavior Management. (2019) 12, 351–360, 10.2147/PRBM.S207283. 31191056 PMC6526330

[72] He Q. , Zhang D. , and Cao S. , Presenteeism and Chinese Clinical Nurses’ Turnover Intention: The Mediating Role of Frustration and Job Burnout, BMC Nursing. (2025) 24, no. 1, 10.1186/s12912-025-03234-2. PMC12135485 40462039

[73] Li Z. , Luo Z. , Chen L. et al., Present Situation and Influencing Factors of Presenteeism Among Nurses in China: A cross-sectional Multicenter Study, BMC Nursing. (2025) 24, no. 1, 10.1186/s12912-025-03095-9. PMC12016474 40269961

