# Supplementary material for: Work–Family Behavioral Role Conflict and Turnover Intention Among Clinical Nurses: Multiple Mediating Effects of Role Overload, Presenteeism, and Psychological Capital
Source: J Nurs Manag. 2026 Aug 13;2026:8648929. doi: 10.1155/jonm/8648929 (PMC13473740; doi:10.1155/jonm/8648929)
Supplement: Supplementary file 1 [file JONM-2026-8648929-s001.docx]

**Supplementary Material**

**Work-Family Behavioral Role Conflict and Turnover Intention Among Clinical Nurses: Multiple Mediating Effects of Role Overload, Presenteeism, and Psychological Capital**

**[Table S1: Descriptive statistics with normality test	2](#_Toc9678)**

**[Table S2: Univariate analysis of turnover intention scores across demographic and professional characteristics of participants (](#_Toc14317)*[N](#_Toc14317)*[=1225)	2](#_Toc14317)**

**[Table S3: Parameter estimates of the direct and indirect paths in the serial multiple mediation model of the relationship between work-family behavioral role conflict and turnover intention (Model 2)	5](#_Toc22559)**

**[Figure S1: First-stage Moderation Models	6](#_Toc27278)**

**[Figure S2: Second-stage Moderation Models	6](#_Toc13853)**

**[Table S4: Standardized Structural Path Coefficients with Moderating Effect of Psychological Capital on Role Overload (Model 5)	7](#_Toc30474)**

**[Table S5: Standardized Structural Path Coefficients with Moderating Effect of Psychological Capital on Presenteeism (Model 6)	8](#_Toc1259)**

**[Table S6: Standardized Structural Path Coefficients with Moderating Effect of Psychological Capital on both Role Overload and Presenteeism (Model 7)	10](#_Toc30464)**

**[Table S7: Standardized structural path coefficients of the parallel multiple mediation model with demographic and work-related covariates controlled (Model 1 with covariates)	11](#_Toc25733)**

**[Table S8: Multiple linear regression analyses of turnover intention using the total WFBRC score and its two sub-dimensions (](#_Toc19414)*[N](#_Toc19414)* [= 1225)	12](#_Toc19414)**

# Table S1: Descriptive statistics with normality test

Table S1 presents the descriptive statistics and normality test results for the study variables. Skewness values ranged from −0.53 to 0.28 and kurtosis values ranged from −0.61 to 1.53, all within acceptable thresholds for normal distribution. The mean scores were as follows: WFBRC (M = 79.57, SD = 21.51), role overload (M = 11.22, SD = 4.04), psychological capital (M = 138.78, SD = 24.74), presenteeism (M = 15.99, SD = 3.89), and turnover intention (M = 9.56, SD = 4.05).

**Table S1: Descriptive statistics with normality test**

| **Variable** | **M** | **Me** | **SD** | **Sk.** | **Kurt.** | **Min** | **Max** |
| --- | --- | --- | --- | --- | --- | --- | --- |
| WFBRC | 79.57 | 83.00 | 21.51 | -0.24 | 0.68 | 30 | 150 |
| Work-to-Family BRC | 44.87 | 45.00 | 12.51 | -0.45 | 0.37 | 15 | 75 |
| Family-to-Work BRC | 34.70 | 35.00 | 11.86 | 0.19 | 0.09 | 15 | 75 |
| Role Overload | 11.22 | 11.00 | 4.04 | 0.14 | -0.61 | 5 | 20 |
| Psychological Capital | 138.78 | 141.00 | 24.74 | -0.53 | 1.53 | 30 | 180 |
| Presenteeism | 15.99 | 16.00 | 3.89 | -0.41 | 0.74 | 6 | 30 |
| Turnover Intention | 9.56 | 9.00 | 4.05 | 0.28 | -0.58 | 4 | 20 |

Note: M, Mean；Me, Median；SD, Standard Deviation；Sk., Skewness；Kurt., Kurtosis；Min, Minimum；Max, Maximum；WFBRC：Work-Family Behavioral Role Conflict；Work-to-Family BRC: Work-to-Family Behavioral Role Conflict；Family-to-Work BRC: Family-to-Work Behavioral Role Conflict.

# Table S2: Univariate analysis of turnover intention scores across demographic and professional characteristics of participants (*N*=1225)

Supplementary Table S2 presents the univariate analysis of turnover intention across demographic and professional characteristics. Significant differences in turnover intention scores were observed across department, age, marital status, parental status, professional title, job function, work experience, average weekly working hours, and average monthly night shifts (all *p* < 0.05). Specifically, higher turnover intention scores were found among nurses working in pediatrics and internal medicine, those aged 26-35 years, those who were unmarried/divorced/widowed, those without children, those with lower professional titles, those working as clinical nurses only, those with shorter work experience, those working more than 40 hours per week, and those with more frequent night shifts. No significant differences were found by gender, education level, employment type, or work stage (all *p* > 0.05).

**Table S2: Univariate analysis of turnover intention scores across demographic and professional characteristics of participants (N=1225)**

| **Variables** | **Turnover Intention score**  **(M±SD)** | ***p*-value** |
| --- | --- | --- |
| Department |  | <0.001 |
| Internal medicine | 10.15±4.05 |  |
| Surgery | 9.91±4.20 |  |
| Gynecology and obstetrics | 9.07±3.57 |  |
| Pediatrics | 10.34±4.09 |  |
| Geriatrics | 10.15±4.16 |  |
| Oncology | 7.91±3.63 |  |
| Comprehensive department | 9.34±3.94 |  |
| Outpatient clinic | 7.34±3.65 |  |
| Emergency department | 9.60±4.03 |  |
| Other | 8.54±3.87 |  |
| Gender |  | 0.940 |
| Male | 9.52 ± 4.26 |  |
| Female | 9.56 ± 4.04 |  |
| Age |  | <0.001 |
| ≤25 years | 9.59 ± 4.28 |  |
| 26-35 years | 9.98 ± 4.05 |  |
| 36-45 years | 9.33 ± 3.94 |  |
| ≥45 years | 7.33 ± 3.44 |  |
| Marital status |  | 0.001 |
| Unmarried, divorced or widowed | 10.12 ± 4.18 |  |
| Married | 9.31 ± 3.98 |  |
| Parental status |  | <0.001 |
| No children | 10.18 ± 4.26 |  |
| With children | 9.20 ± 3.89 |  |
| Education level |  | 0.809 |
| Bachelor's degree | 9.56 ± 4.07 |  |
| Master's degree or above | 9.44 ± 3.87 |  |
| Professional title |  | 0.003 |
| Junior nurse | 9.72 ± 4.25 |  |
| Senior nurse | 9.83 ± 4.01 |  |
| Nurse-in-charge | 9.48 ± 4.01 |  |
| Assistant chief senior nurse and above | 7.57 ± 4.04 |  |
| Job function |  | 0.024 |
| Clinical nurse | 9.72 ± 4.08 |  |
| Clinical nurse with additional administrative/teaching/research duties | 9.13 ± 3.96 |  |
| Employment type |  | 0.551 |
| permanent staff | 9.41 ± 4.12 |  |
| contract staff | 9.59 ± 4.04 |  |
| Work stage |  | 0.112 |
| Fixed in a specific department | 9.62 ± 4.07 |  |
| Rotation | 9.01 ± 3.87 |  |
| Work experience (years) |  | <0.001 |
| ≤5 | 10.13 ± 4.20 |  |
| 6-10 | 9.81 ± 3.92 |  |
| 11-15 | 9.59 ± 3.99 |  |
| >15 | 7.79 ± 3.67 |  |
| Average weekly working hours (hours) |  | <0.001 |
| ≤40 | 9.03 ± 3.75 |  |
| >40 | 10.37 ± 4.37 |  |
| Average monthly night shifts |  | <0.001 |
| 0 | 8.13 ± 3.77 |  |
| 1-4 | 8.72 ± 3.83 |  |
| 5-8 | 10.08 ± 4.09 |  |
| >8 | 10.09 ± 3.97 |  |

# Table S3: Parameter estimates of the direct and indirect paths in the serial multiple mediation model of the relationship between work-family behavioral role conflict and turnover intention (Model 2)

Table S3 summarizes the parameter estimates for the serial multiple mediation model (Model 2) (model fit indices: χ²/df = 4.935, CFI = 0.975, TLI = 0.901, and RMSEA = 0.071, SRMR = 0.038 ). The total effect of WFBRC on turnover intention was significant (*β* = 0.612, 95% CI [0.569, 0.661]), and the direct effect remained significant after including mediators (*β* = 0.261, 95% CI [0.208, 0.316]). The total indirect effect was also significant (*β* = 0.352, 95% CI [0.311, 0.399]). Regarding direct paths, WFBRC was significantly associated with role overload (*β* = 0.584), PsyCap (*β* = −0.427), and presenteeism (*β* = 0.329). Role overload showed significant associations with presenteeism (*β* = 0.300) and turnover intention (*β* = 0.354). PsyCap (*β* = −0.098) and presenteeism (*β* = 0.208) were each significantly related to turnover intention. All specific indirect effects were significant. These included the paths through role overload (*β* = 0.206), through presenteeism (*β* = 0.068), through PsyCap (*β* = 0.042), and the sequential pathway via role overload and presenteeism (*β* = 0.036). All confidence intervals excluded zero.

**Table S3: Parameter estimates of the direct and indirect paths in the serial multiple mediation model of the relationship between work-family behavioral role conflict and turnover intention (Model 2)**

| **Structural Path** | ***β*** | **95% CI** |
| --- | --- | --- |
| **Total Effect** | 0.612*** | [0.569, 0.661] |
| **Direct Effect** (WFBRC → Turnover Intention) | 0.261*** | [0.208, 0.316] |
| **Total Indirect Effect** | 0.352*** | [0.311, 0.399] |
| Direct paths |  |  |
| WFBRC → Role Overload | 0.584*** | [0.538, 0.623] |
| WFBRC → PsyCap | -0.427*** | [-0.498, -0.350] |
| WFBRC → Presenteeism | 0.329*** | [0.276, 0.384] |
| Role Overload → Presenteeism | 0.300*** | [0.241, 0.361] |
| Role Overload → Turnover Intention | 0.354*** | [0.297, 0.407] |
| PsyCap → Turnover Intention | -0.098*** | [-0.151, -0.049] |
| Presenteeism → Turnover Intention | 0.208*** | [0.151, 0.262] |
| **Indirect Effects** (Mediation Paths) |  |  |
| WFBRC → Role Overload → Turnover Intention | 0.206*** | [0.176, 0.241] |
| WFBRC → Presenteeism → Turnover Intention | 0.068*** | [0.049, 0.09] |
| WFBRC → PsyCap → Turnover Intention | 0.042*** | [0.021, 0.065] |
| WFBRC → Role Overload → Presenteeism → Turnover Intention | 0.036*** | [0.026, 0.049] |

Note: ****p* < 0.001. *β* = Standardized regression coefficient; CI = bias-corrected bootstrap confidence interval. Effects were considered significant when the 95% CI did not include zero.

# Figure S1: First-stage Moderation Models

Figure S1 present the first-stage moderation models, PsyCap did not significantly moderate the association between WFBRC and role overload (*p* = 0.529, Model 3) (model fit indices: χ²/df = 9.771, CFI = 0.922, TLI = 0.765, RMSEA = 0.108, SRMR = 0.069) or between WFBRC and presenteeism (*p* = 0.921, Model 4) (model fit indices: χ²/df = 6.674, CFI = 0.928, TLI = 0.844, RMSEA = 0.145, SRMR = 0.055).

**Figure S1: First-stage Moderation Models**

Note: *** *p* <0.001. Statistical significance was determined based on 95 % bias-corrected bootstrap confidence intervals. Effects are considered significant if the confidence interval does not include zero.

# Figure S2: Second-stage Moderation Models

Figure S2 present second-stage moderation models, significant interaction effects were observed. PsyCap moderated the relationship between role overload and turnover intention (*β* = −0.058, p = 0.020; Model 5) (model fit indices: χ²/df = 4.574, CFI = 0.956, TLI = 0.905, RMSEA = 0.095, SRMR = 0.043), as well as the association between presenteeism and turnover intention (*β* = −0.069, p = 0.004; Model 6) (model fit indices: χ²/df = 5.472, CFI = 0.922, TLI = 0.869, RMSEA = 0.098, SRMR = 0.067). When both second-stage moderation paths were simultaneously entered into Model 7 (χ²/df = 6.293, CFI = 0.915, TLI = 0.855, RMSEA = 0.106, SRMR = 0.061), neither interaction remained statistically significant.

**Figure S2: Second-stage Moderation Models**

Note: ***p* <0.01, *** *p* <0.001.

# Table S4: Standardized Structural Path Coefficients with Moderating Effect of Psychological Capital on Role Overload (Model 5)

Table S4 presents the standardized structural path coefficients for Model 5, which tested the moderating effect of psychological capital on the pathway from role overload to turnover intention. The total effect of WFBRC on turnover intention was significant (*β* = 0.591, 95% CI [0.546, 0.636]), and the direct effect remained significant (*β* = 0.269, 95% CI [0.216, 0.323]). For the direct paths, WFBRC showed significant associations with role overload (*β* = 0.584) and presenteeism (*β* = 0.504). Role overload (*β* = 0.363), presenteeism (*β* = 0.210), and psychological capital (*β* = −0.113) were each significantly related to turnover intention. A significant moderating effect of psychological capital was found on the relationship between role overload and turnover intention (*β* = −0.058, 95% CI [−0.108, −0.012]). Regarding indirect effects, the pathway from WFBRC to turnover intention via role overload was significant under both low PsyCap (*β* = 0.192) and high PsyCap (*β* = 0.148) conditions. The indirect effect via presenteeism was also significant (*β* = 0.106). The total indirect effect was significant (*β* = 0.324, 95% CI [0.282, 0.368]). All confidence intervals excluded zero.

**Table S4: Standardized Structural Path Coefficients with Moderating Effect of Psychological Capital on Role Overload (Model 5)**

| **Structural Path** | ***β*** | **95% CI** |
| --- | --- | --- |
| **Total Effect** | 0.591*** | [0.546, 0.636] |
| **Direct Effect** (WFBRC → Turnover Intention) | 0.269*** | [0.216, 0.323] |
| Direct Paths |  |  |
| WFBRC → Role Overload | 0.584*** | [0.538, 0.623] |
| WFBRC → Presenteeism | 0.504*** | [0.464, 0.542] |
| Role Overload → Turnover Intention | 0.363*** | [0.305, 0.417] |
| Presenteeism → Turnover Intention | 0.210*** | [0.152, 0.266] |
| PsyCap → Turnover Intention | -0.113*** | [-0.169, -0.059] |
| Moderating Effect |  |  |
| Role Overload × PsyCap → Turnover Intention | -0.058* | [-0.108, -0.012] |
| **Indirect Effects** |  |  |
| WFBRC → Role Overload → Turnover Intention (Low PsyCap) | 0.192*** | [0.174, 0.247] |
| WFBRC → Role Overload → Turnover Intention (High PsyCap) | 0.148*** | [0.126, 0.198] |
| WFBRC → Presenteeism → Turnover Intention | 0.106*** | [0.083, 0.152] |
| **Total Indirect Effect** | 0.324*** | [0.282, 0.368] |

Note: ***p < 0.001. β = Standardized regression coefficient; CI = bias-corrected bootstrap confidence interval. Effects were considered significant when the 95% CI did not include zero. Low PsyCap condition represents psychological capital at 1 standard deviation below the mean; High PsyCap condition represents psychological capital at 1 standard deviation above the mean.

# Table S5: Standardized Structural Path Coefficients with Moderating Effect of Psychological Capital on Presenteeism (Model 6)

Table S5 summarizes the standardized structural path coefficients for Model 6, which examined the moderating effect of psychological capital on the pathway from presenteeism to turnover intention. The total effect of WFBRC on turnover intention was significant (*β* = 0.591, 95% CI [0.546, 0.636]), and the direct effect remained significant (*β* = 0.266, 95% CI [0.212, 0.319]). The total indirect effect was also significant (*β* = 0.324, 95% CI [0.282, 0.368]). For the direct paths, WFBRC was significantly associated with both role overload (*β* = 0.584) and presenteeism (*β* = 0.504). Role overload (*β* = 0.354), presenteeism (*β* = 0.237), and psychological capital (*β* = −0.103) were each significantly related to turnover intention. A significant moderating effect was observed for psychological capital on the relationship between presenteeism and turnover intention (*β* = −0.069, 95% CI [−0.119, −0.024]). For the indirect effects, the pathway from WFBRC to turnover intention via role overload was significant (*β* = 0.207). The indirect effect via presenteeism was significant under both low PsyCap (*β* = 0.119) and high PsyCap (*β* = 0.068) conditions. All confidence intervals excluded zero.

**Table S5: Standardized Structural Path Coefficients with Moderating Effect of Psychological Capital on Presenteeism (Model 6)**

| **Structural Path** | ***β*** | **95% CI** |
| --- | --- | --- |
| **Total Effect** | 0.591*** | [0.546, 0.636] |
| **Direct Effect**（WFBRC → Turnover Intention） | 0.266*** | [0.212, 0.319] |
| Direct Paths |  |  |
| WFBRC → Role Overload | 0.584*** | [0.538, 0.623] |
| WFBRC → Presenteeism | 0.504*** | [0.464, 0.542] |
| Role Overload → Turnover Intention | 0.354*** | [0.294, 0.408] |
| Presenteeism → Turnover Intention | 0.237*** | [0.180, 0.296] |
| PsyCap → Turnover Intention | -0.103*** | [-0.154, -0.053] |
| **Moderating Effect** |  |  |
| Presenteeism × Psychological Capital → Turnover Intention | -0.069** | [-0.119, -0.024] |
| **Indirect Effects** |  |  |
| WFBRC → Role Overload → Turnover Intention | 0.207*** | [0.174, 0.247] |
| WFBRC → Presenteeism → Turnover Intention (Low PsyCap) | 0.119*** | [0.087, 0.156] |
| WFBRC → Presenteeism → Turnover Intention (High PsyCap) | 0.068*** | [0.049, 0.090] |
| **Total Indirect Effect** | 0.324*** | [0.282, 0.368] |

Note: ****p* < 0.001. *β* = Standardized regression coefficient; CI = bias-corrected bootstrap confidence interval. Effects were considered significant when the 95% CI did not include zero. Low PsyCap condition represents psychological capital at 1 standard deviation below the mean; High PsyCap condition represents psychological capital at 1 standard deviation above the mean.

# Table S6: Standardized Structural Path Coefficients with Moderating Effect of Psychological Capital on both Role Overload and Presenteeism (Model 7)

Table S6 presents the standardized structural path coefficients for Model 7, which examined the simultaneous moderating effects of psychological capital on both role overload and presenteeism. The moderating effects of psychological capital on the role overload to turnover intention path (*β* = −0.030, 95% CI [−0.089, 0.029]) and the presenteeism to turnover intention path (*β* = −0.052, 95% CI [−0.112, 0.006]) were not statistically significant, as their confidence intervals included zero.

**Table S6: Standardized Structural Path Coefficients with Moderating Effect of Psychological Capital on both Role Overload and Presenteeism (Model 7)**

| **Structural Path** | ***β*** | **95% CI** |
| --- | --- | --- |
| **Total Effect** | 0.591*** | [0.546, 0.636] |
| **Direct Effect**（WFBRC → Turnover Intention） | 0.267*** | [0.214, 0.321] |
| **Total Indirect Effect** | 0.324*** | [0.282, 0.368] |
| Direct paths |  |  |
| WFBRC → Role Overload | 0.584*** | [0.538, 0.623] |
| WFBRC → Presenteeism | 0.504*** | [0.464, 0.542] |
| Role Overload → Turnover Intention | 0.357*** | [0.298, 0.412] |
| Presenteeism → Turnover Intention | 0.230*** | [0.168, 0.292] |
| PsyCap → Turnover Intention | -0.108*** | [-0.164, -0.053] |
| Indirect Effects (Mediation Paths) |  |  |
| WFBRC → Role Overload → Turnover Intention | 0.208*** | [0.174, 0.247] |
| WFBRC → Presenteeism → Turnover Intention | 0.116*** | [0.083, 0.152] |
| **Moderating Effects** |  |  |
| Role Overload × PsyCap → Turnover Intention | -0.030 | [-0.089, 0.029] |
| Presenteeism × PsyCap → Turnover Intention | -0.052 | [-0.112, 0.006] |

Note: ****p* < 0.001. *β* = Standardized regression coefficient; CI = bias-corrected bootstrap confidence interval. Effects were considered significant when the 95% CI did not include zero.

# Table S7: Standardized structural path coefficients of the parallel multiple mediation model with demographic and work-related covariates controlled (Model 1 with covariates)

Table S7 reports the standardized structural path coefficients for the parallel multiple mediation model with demographic and work-related covariates controlled(model fit indices: χ²/df = 4.781 , CFI = 0.914, TLI = 0.898, RMSEA = 0.072, and SRMR = 0.041). The total effect of WFBRC on turnover intention remained significant (*β* = 0.595, 95% CI [0.567, 0.657]), as did the direct effect (*β* = 0.273, 95% CI [0.219, 0.328]). The total indirect effect was also significant (*β* = 0.322, 95% CI [0.315, 0.392]). For the direct mediation paths, WFBRC was positively associated with role overload (*β* = 0.561) and presenteeism (*β* = 0.501), and negatively associated with psychological capital (*β* = −0.428). Role overload (*β* = 0.343), presenteeism (*β* = 0.193), and psychological capital (*β* = −0.077) were each significantly associated with turnover intention. Three indirect pathways were significant: WFBRC → role overload → turnover intention (*β* = 0.192, 95% CI [0.174, 0.247]), WFBRC → presenteeism → turnover intention (*β* = 0.097, 95% CI [0.083, 0.152]), WFBRC → PsyCap → turnover intention (*β* = 0.033, 95% CI [0.016, 0.074]). All confidence intervals excluded zero, indicating statistically significant mediation effects.

**Table S7: Standardized structural path coefficients of the parallel multiple mediation model with demographic and work-related covariates controlled (Model 1 with covariates)**

| **Structural Paths** | ***β*** | **95% CI** |
| --- | --- | --- |
| **Total Effect** | 0.595*** | [0.567, 0.657] |
| **Direct Effect**（WFBRC → Turnover Intention） | 0.273*** | [0.219, 0.328] |
| **Total Indirect Effect** | 0.322*** | [0.315, 0.392] |
| Direct paths |  |  |
| WFBRC → Role Overload | 0.561*** | [0.512, 0.603] |
| WFBRC → PsyCap | -0.428*** | [-0.503, -0.348] |
| WFBRC → Presenteeism | 0.501*** | [0.459, 0.540] |
| Role Overload → Turnover Intention | 0.343*** | [0.286, 0.396] |
| PsyCap → Turnover Intention | -0.077** | [-0.128, -0.029] |
| Presenteeism → Turnover Intention | 0.193*** | [0.138, 0.248] |
| **Indirect Effects** |  |  |
| WFBRC → Role Overload → Turnover Intention | 0.192*** | [0.174, 0.247] |
| WFBRC → Presenteeism → Turnover Intention | 0.097*** | [0.083, 0.152] |
| WFBRC → PsyCap → Turnover Intention | 0.033** | [0.016, 0.074] |

Note: *** *p* <0.001; ** *p* <0.01; Model 1 controls for demographic and work-related covariates; *β* = Standardized regression coefficient; CI = bias-corrected bootstrap confidence interval. Effects were considered significant when the 95% CI did not include zero.

# Table S8: Multiple linear regression analyses of turnover intention using the total WFBRC score and its two sub-dimensions (*N* = 1225)

Table S8 reports the supplementary multiple linear regression analyses of turnover intention using the total WFBRC score and its two sub-dimensions. In Model a, the total WFBRC score was positively associated with turnover intention (*β* = 0.600, *p* < 0.001). In Model b, when the two sub-dimensions were entered simultaneously, both family-to-work BRC (*β* = 0.400, *p* < 0.001) and work-to-family BRC(*β* = 0.279, *p* < 0.001) were positively associated with turnover intention.

**Table S8: Multiple linear regression analyses of turnover intention using the total WFBRC score and its two sub-dimensions (N = 1225)**

| **Variables** | ***B*** | **SE** | ***β*** | **t** | ***p*** | ***VIF*** |
| --- | --- | --- | --- | --- | --- | --- |
| **Model a** |  |  |  |  |  |  |
| Constant | 0.469 | 0.639 |  | 0.733 | 0.463 |  |
| WFBRC total score | 0.113 | 0.004 | 0.600 | 27.033 | <0.001 | 1.034 |
| Age | -0.433 | 0.148 | -0.081 | -2.922 | 0.004 | 1.624 |
| Average monthly night shifts | 0.429 | 0.108 | 0.093 | 3.984 | <0.001 | 1.150 |
| Average weekly working hours | 0.573 | 0.184 | 0.069 | 3.115 | 0.002 | 1.033 |
| Department(Geriatrics) | 0.967 | 0.346 | 0.061 | 2.792 | 0.005 | 1.011 |
| Department(Oncology) | -1.072 | 0.391 | -0.060 | -2.744 | 0.006 | 1.008 |
| Parental status | -0.517 | 0.226 | -0.061 | -2.290 | 0.022 | 1.503 |
| **Model b** |  |  |  |  |  |  |
| Constant | 0.340 | 0.639 |  | 0.533 | 0.594 |  |
| Family-to-work BRC | 0.137 | 0.009 | 0.400 | 15.110 | <0.001 | 1.481 |
| Work-to-family BRC | 0.090 | 0.009 | 0.279 | 10.299 | <0.001 | 1.546 |
| Age | -0.398 | 0.148 | -0.075 | -2.687 | 0.007 | 1.634 |
| Average monthly night shifts | 0.453 | 0.108 | 0.099 | 4.213 | <0.001 | 1.157 |
| Average weekly working hours | 0.626 | 0.184 | 0.075 | 3.397 | 0.001 | 1.043 |
| Department(Geriatrics) | 0.974 | 0.345 | 0.062 | 2.822 | 0.005 | 1.011 |
| Department(Oncology) | -1.053 | 0.389 | -0.059 | -2.704 | 0.007 | 1.008 |
| Parental status | -0.453 | 0.226 | -0.054 | -2.005 | 0.045 | 1.517 |

Notes:

1. WFBRC = work-family behavioral role conflict.

2. Model a included the total WFBRC score. Model b replaced the total WFBRC score with the two sub-dimensions(Family-to-work BRC and Work-to-family BRC) of WFBRC. Both models included department, age, marital status, parental status, professional title, job function, work experience, average monthly night shifts, and average weekly working hours.

3. For Model 1, *R* = 0.648, *R²* = 0.420, adjusted *R²* = 0.417, *F* = 126.059, *p* < 0.001, and Durbin-Watson = 2.013. For Model 2, *R* = 0.651, *R²* = 0.424, adjusted *R²* = 0.421, *F* = 112.088, *p* < 0.001, and Durbin-Watson = 2.016.
